# Molecular genetics of chronic neutrophilic leukemia, chronic myelomonocytic leukemia and atypical chronic myeloid leukemia

**DOI:** 10.1186/s13045-014-0093-1

**Published:** 2014-12-12

**Authors:** Bing Li, Robert Peter Gale, Zhijian Xiao

**Affiliations:** MDS and MPN Centre, Institute of Hematology and Blood Diseases Hospital, Chinese Academy of Medical Sciences & Peking Union Medical College, 288 Nanjing Road, Tianjin, 300020 China; State Key Laboratory of Experimental Hematology, Institute of Hematology and Blood Diseases Hospital, Chinese Academy of Medical Sciences & Peking Union Medical College, Tianjin, China; Hematology Research Centre, Division of Experimental Medicine, Department of Medicine, Imperial College London, London, UK

**Keywords:** Molecular genetics, Chronic neutrophilic leukemia, Chronic myelomonocytic leukemia, Atypical chronic myeloid leukemia

## Abstract

According to the 2008 World Health Organization classification, chronic neutrophilic leukemia, chronic myelomonocytic leukemia and atypical chronic myeloid leukemia are rare diseases. The remarkable progress in our understanding of the molecular genetics of myeloproliferative neoplasms and myelodysplastic/myeloproliferative neoplasms has made it clear that there are some specific genetic abnormalities in these 3 rare diseases. At the same time, there is considerable overlap among these disorders at the molecular level. The various combinations of genetic abnormalities indicate a multi-step pathogenesis, which likely contributes to the marked clinical heterogeneity of these disorders. This review focuses on the current knowledge and challenges related to the molecular pathogenesis of chronic neutrophilic leukemia, chronic myelomonocytic leukemia and atypical chronic myeloid leukemia and relationships between molecular findings, clinical features and prognosis.

## Introduction

Chronic neutrophilic leukemia (CNL), chronic myelomonocytic leukemia (CMML) and atypical chronic myeloid leukemia (CML) are rare diseases categorized into the 2008 World Health Organization (WHO) as either myeloproliferative neoplasms (MPN; CNL) or myelodysplastic/myeloproliferative neoplasms (MDS/MPN; CMML and a CML). These diseases share several characteristics including an enlarged liver and spleen, an increased WBC and a hyper-cellular bone marrow. They are distinguished from each other based on numbers and types of leukemia cells in the blood, appearance of leukemia cells in the blood and bone marrow cells and cytogenetic and molecular abnormalities including absence of t(9:22) and BCR/ABL1 and rearrangement of platelet-derived growth factor receptor, alpha polypeptide (PDGFRA), platelet-derived growth factor receptor, beta polypeptide (PDGFRB) or fibroblast growth factor receptor 1 (FGFR1). Previously, it was sometimes difficult to distinguish these disease from each other and from other MPNs including chronic myeloid leukemia (CML), polycythaemia vera (PV), essential thrombocythaemia (ET) and primary myelofibrosis (PMF) which have different molecular abnormalities including mutations in Janus kinase 2 (JAK2), calreticulin (CALR) and MPL proto-oncogene, thrombopoietin receptor (MPL). Molecular characterization of CNL, CMML and atypical CML has advanced even further with the use of next generation sequencing. Important genetic abnormalities have been identified which help to distinguish between these diseases. This review focuses on the current knowledge and challenges related to the molecular pathogenesis of CNL, CMML and atypical CML and relationships between molecular findings, clinical features and prognosis.

### Molecular genetics of CNL

#### Colony stimulating factor 3 receptor (CSF3R) mutations

**Figure 1:**
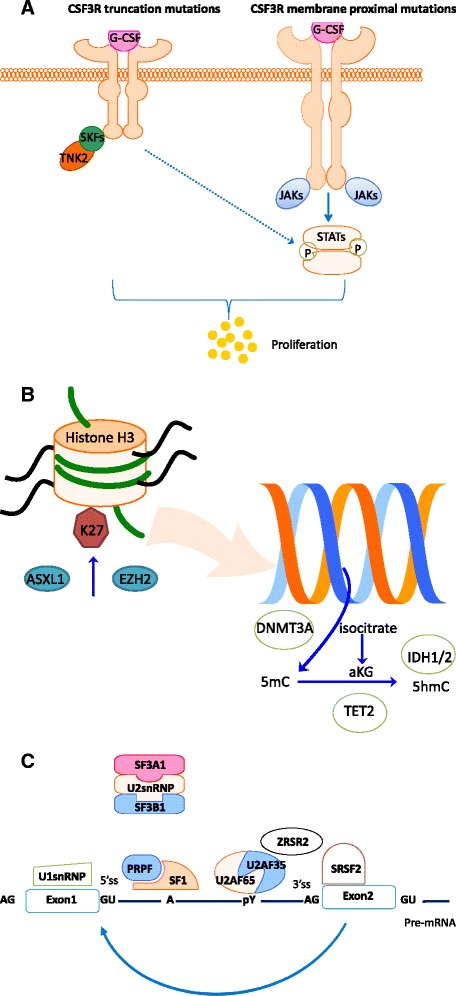
**Somatic mutations affect genes involved in CSF3R (A), epigenetic regulation (B) and RNA splicing (C).**

In 2013, Maxson *et al*. reported frequent mutations in CSF3R in patients with CNL and atypical CML [1]. Such mutations are rare in patients with AML. Two types of mutations were found. CSF3R^T618I^ and CSF3R^T615A^ are most common mutations, which are in the membrane proximal region which mediates proliferative and survival signals. These mutations occur alone or with another nonsense mutations truncating the cytoplasmic tail of the coding region important in transduction of maturation and suppression of proliferation [1,2]. Membrane proximal mutations result in activation of JAK signaling pathways and truncation mutations in preferential activation of SRC family–TNK2 kinase signaling (Figure 1A) [1]. The aetiological role of CSF3R^T618I^ mutation in these diseases was studied in a model in which mice transplanted with CSF3R^T618I^–expressing hematopoietic cells. These mice developed a CNL-like phenotype characterized by hyper-cellular bone marrow and granulocyte infiltrates in the spleen and liver and died [3].

Pardanani *et al.* [4] sequenced the implicated exons of CSF3R in 35 suspected subjects with CNL, 12 subjects with *confirmed* CNL and 6 subjects with CNL and a monoclonal gammopathy or lymphoid neoplasm. All subjects who fulfilled the 2008 WHO criteria for CNL had a CSF3R proximal membrane mutation, most frequently CSF3R^T618I^. One subject had CSF3R^M696T^ and 1, CSF3R^I598I^. One of these subjects also had a truncating CSF3R mutation. No one with WHO-defined CNL but with a monoclonal gammopathy or lymphoid neoplasm had a CSF3R mutation. These data explain the different bases and prognoses between CNL with and without a monoclonal gammopathy [5-8]. Our study in Chinese with WHO-defined CNL confirmed these findings [9]. We found a CSF3R^T618I^ mutation in all subjects with CNL without a monoclonal gammopathy but not in 2 subjects with features of CNL with a monoclonal gammopathy. These data indicate CSF3R mutations are a highly sensitive and specific molecular marker for WHO-defined CNL and we suggest it be included into the revised criteria.

#### JAK2 ^V617F^

JAK2 ^V617F^ is common in patients with BCR/ABL1 negative MPNs and is rare in patients with CML but not in those with lymphoid neoplasms, reactive myelo-proliferative disorders or normals [10,11]. About 13 patients with CNL with JAK2 ^V617F^ are reported [12-20]. These patients had typical features of CNL without the erythrocytosis, thrombocytosis and bone marrow fibrosis typical of PV, ET and PMF, but CSF3R mutations were not tested. Other recent studies report no JAK2 ^V617F^ in patients with CNL with CSF3R mutations [4,9]. Also controversial is whether patients with CNL and JAK2 ^V617F^ have a different prognosis than patients with CNL without JAK2 ^V617F^. Some data suggest CNL with JAK2^V617F^ mutation has an indolent course with long intervals of stable disease on hydroxyurea [12]. Other data suggest person with CNL and JAK2^V617F^ have a worse prognosis and a high risk of transformation to AML [13,14]. The underlying issue, of course, is whether the current WHO-criteria are sufficient to accurately diagnose CNL. Do patients with *seeming* CNL but with JAK2^V617F^ rather than a CSF3R mutation really have CNL or a different disorder not currently recognized in the WHO-criteria. This distinction is arbitrary but we favor reserving the CNL-designation in future revisions of the WHO-criteria for patients with CSF3R mutations. This would, of course, require a new designator for patients with a clinical phenotype resembling CNL but with JAK2^V617F^. This situation is distinct than those in which a person with a CNL phenotype and a CSF3R mutation can also have a 2^nd^ mutation such as SETBP1 (see below).

#### SET binding protein 1 (SETBP1) mutations

Three small studies reported SETBP1 mutations in some patients with CNL [4,9,21]. Pardanani *et al.* [4] reported 6 of 34 patients with clinically-suspected CNL had SETBP1 mutations and 4 of 12 patients with WHO-defined CNL had SETBP1 mutation and CSF3R^T618I^. We found SETBP1 mutations in 6 of 8 patients with CNL with CSF3R^T618I^ [9]. Piazza *et al.* [21] reported a SETBP1 mutation in 4 patients with CNL and a CSF3R mutation. These patients had a worse prognosis than those with CNL without a SETBP1 mutation [4,21,22], an observation requires confirmation.

### Molecular genetics of CMML

Mutations are detected in about 90 percent of patients with CMML [23-25]. These mutations are clustered into four categories: (1) mutations involving epigenetic regulator genes (Figure 1B) [23-26]; (2) mutations involving the spliceosome component pathway (Figure 1C) [27-30]; (3) mutations involving transcription factors [24,31-33]; and (4) mutations involving signaling regulator genes [24,26,34-37].

#### Epigenetic regulator gene mutations

Mutations in tet methylcytosine dioxygenase 2 (TET2) mutations are detected in 40–60 percent of patients with CMML [24,26,38,39]. There is controversy whether TET2 mutations in patients with CMML is associated with prognosis. Kosmider *et al.* [38] reported TET2 mutation was associated with a poor prognosis but this was not confirmed in 2 recent large series [24,30]. Mutations in DNA (cytosine-5-)-methylthransferase 3 alpha (DNMT3A) and isocitrate dehydrogenase 1/2 (IDH1/2) are rare in CMML [24,26,40]. DNMT3A mutations trended to occur in CMML-2 and were reported in 6 of 20 patients with CMML-derived AML [26]. Additional sex combs like transcriptional regulator 1 (ASXL1) mutations are detected in 40–50 percent of patients with CMML [24,30,41] who are reported to have a higher WBC and higher levels of monocytes and immature myeloid cells in the blood [24,30]. Clinical features of patients with CMML and c.1934dupG;p.G646WfsX12 are similar to those of patients with other ASXL1 mutations [24,30]. Conclusions from 2 large series about prognostic impact of ASXL1 mutations on survival are contradictory. Itzykson *et al.* reported ASXL1 mutation was an unfavorable independent prognostic factor [24]. However, data from Patnaik *et al.* showed that ASXL1 mutations had no impact on survival [30]. This controversy requires resolution. Enhancer of zeste 2 polycomb repressive complex 2 subunit (EZH2) mutations are uncommon in CMML [24].

#### Spliceosome component mutations

About 60 percent of patients with CMML have mutations in genes involved in RNA-splicing [24]. Most common are mutations in serine/arginine-rich splicing factor 2 (SRSF2) in 30–50 percent of cases. These mutations are associated with increased age, less pronounced anemia and a diploid karyotype but not with prognosis [24,28,29,42]. SRSF2 or zinc finger (CCCH type), RNA-binding motif and serine/arginine rich 2 (ZRSR2) mutations are frequently concordant with TET2 mutations [24,28,43].

#### Mutations affecting transcription

Mutations in the runt-related transcription factor 1 (RUNX1) transcription factor are detected in 15–40 percent of patients with CMML [24,31-33]. Patients with CMML and RUNX1 mutations may have a higher risk of transformation to AML [31,33]. CCAAT/enhancer binding protein, alpha (CEBPA) mutations are rare in patients with CMML [33].

#### Signaling regulator gene mutations

Kosmider *et al.* [35] reported variant CSF3R somatic mutations in about 4 percent of patients with CMML with high concordance for ASXL1 mutations. These patients were reported to have a poor prognosis. SETBP1 mutations are detected in about 5 percent of patients with CMML and are associated with a poor prognosis in patients who also have an ASXL1 mutation [35-37]. SETBP1 and CSF3R mutations seem mutually exclusive in CMML. Cbl proto-oncogene, E3 ubiquitin protein ligase (CBL) mutations are detected in about 10 percent of patients with CMML [24,34,44,45].

Recently, Itzykson *et al.* tracked mutations in single-cell-derived myeloid colonies after *in vitro* culture in patients with CMML [25]. Their data indicate a preferential order of mutation acquisition in progenitor cells. Mutations in TET2 (or IDH1 and IDH2) or ASXL1 occur 1^st^ followed by mutations in SRSF2 or spliceosome component pathway genes followed by mutations in signal transduction-related genes [25]. These data recall data from studies of the relationship between temporal acquisition of mutations of JAK2^V617F^ and TET2 or ASXL1 in patients with other MPNs where clinical and laboratory features are correlated with which mutation occurs 1^st^ [46,47].

### Molecular genetics of atypical CML

#### SETBP1 mutations

SETBP1 mutations are detected in about 25–30 percent of patients with atypical CML [21,48]. These patients have higher WBCs and a worse prognosis than patients without SETBP1 mutations [21]. SETBP1 mutations are associated with del(7) and iso(17)(q10)), ASXL1 and CBL mutations are exclusive of JAK2 and TET2 mutations in patients with atypical CML [48]. Recent study [49] showed that overexpression of SETBP1 associated with micro-RNA desregulation in MPN.

#### Other mutations

CSF3R mutations do not occur in patients with atypical CML [4]. In contrast, TET2 mutations are common [23,37,48,50]. ASXL1 mutations [20,51] are detected in 20–70 percent of patients with atypical CML and SRSF2 mutations about 40 percent [51]. SRFS2, SETBP1 and ASXL1 mutations are often confounded [51].

### Next-generation sequencing (NGS) in MPN and MDS/MPN

NGS technology, such as whole-exons sequencing (WES) has demonstrated its power in re-sequecing human genomes to enhance our understanding of how molecular genetics affect MPN and MDS/MPN [1,15,16,21,25]. However, the challenge is to provide genetic information in a timely and affordable way. Thus, the utility of detailed targeted resequencing by capturing technologies is currently reasonable in clinical practicality. Consequently, molecular biomarkers will soon no longer be sequenced individually. Instead, panels of biomarkers will be assessed in a massively parallel way with high sensitivity and multiplexing in patients with MPN or MDS/MPN for diagnosis and individualized treatment regimens.

## Conclusion

**Figure 2:**
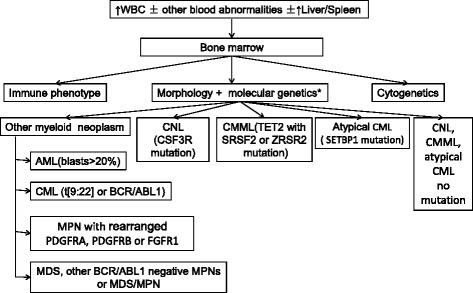
**A schematic approach, outlining the evaluation for a patient presenting with peripheral blood leukocytosis.** * Molecular genetics test should include mutations involved JAK2, CALR, MPL, CSF3R, TET2, SRSF2, ZRSR2, ASXL1, SETBP1 and BCR-ABL1 fusion genes and rearrangement of PDGFRA, PDGFRB or FGFR1. AML, Acute myeloid leukaemia; CML, chronic myelogenous leukemia; CNL,Chronic neutrophilic leukemia; CMML, chronic myelomonocytic leukemia; MDS, myelodysplastic syndromes; MPN, myeloproliferative neoplasms; MDS/MPN, myelodysplastic/myeloproliferative neoplasms.

The mutational landscapes or terrains of CNL, CMML and atypical CML are complex, but some mutations are relatively specific in these 3 disorders. Furthermore, the order in which mutations are acquired may correlate with clinical features and prognosis. Therefore, analyse of mutations in MPNs is increasingly important for several reasons. 1^st^, mutation analyses is useful in trying to determine the aetiology of an increased WBC. For example, mutations in CSF3R suggest CNL, SETBP1 mutations, atypical CML and concurrent TET2 and SRSF2 or ZRSR2 mutations, CMML. Analyses of these frequently mutated genes can determine clonality and distinguish neoplasms from non-neoplastic increases in the WBC (Figure 2). 2^nd^, mutations in ASXL1, SEBP1, DNMT3A are associated, albeit inconsistently, with a poor prognosis in MPNs and MDS/MPNs. These data, if confirmed, could be added to prognostic scoring systems. 3^rd^, identifying specific molecular abnormalities can suggest new therapy targets for these diseases, such as hedgehog pathway inhibitors [52].
